# Comparison of a Novel Temperature-Controlled Diamond-Tip Catheter and a Power-Controlled Gold-Tip Catheter for the Irrigated Ablation of Cavotricuspid Isthmus-Dependent Atrial Flutter

**DOI:** 10.3390/jcm14030701

**Published:** 2025-01-22

**Authors:** Eric Auf der Maur, Thomas Kueffer, Gregor Thalmann, Nikola A. Kozhuharov, Oskar Galuszka, Salik ur Rehman Iqbal, Antonio Madaffari, Helge Servatius, Andreas Haeberlin, Fabian Noti, Hildegard Tanner, Laurent Roten, Tobias Reichlin

**Affiliations:** Department of Cardiology, Inselspital, Bern University Hospital, University of Bern, 3012 Bern, Switzerlandthomas.kueffer@insel.ch (T.K.); gregor.thalmann@insel.ch (G.T.); nikola.kozhuharov@insel.ch (N.A.K.); oskar.galuszka@insel.ch (O.G.); salikurrehman.iqbal@insel.ch (S.u.R.I.); antonio.madaffari@apss.tn.it (A.M.); helge.servatius@insel.ch (H.S.); andreas.haeberlin@insel.ch (A.H.); fabian.noti@insel.ch (F.N.); hildegard.tanner@insel.ch (H.T.); laurent.roten@insel.ch (L.R.)

**Keywords:** atrial flutter, ablation, catheter, cavotricuspid isthmus

## Abstract

**Background/Objectives**: Radiofrequency (RF) ablation of the cavotricuspid isthmus (CTI) is a recommended treatment option for typical atrial flutter (AFL). While power-controlled ablation has been the current standard, a novel temperature-controlled ablation system has been introduced. We aimed to compare the procedural efficacy and one-year outcome of a temperature-controlled diamond-tip catheter with an established power-controlled gold-tip catheter. **Methods**: Consecutive patients undergoing ablation of CTI-dependent AFL using a power-controlled catheter or the novel temperature-controlled catheter were enrolled. Patients were followed up using a 7-day electrocardiogram after 3, 6, and 12 months. The primary endpoint was acute efficacy (procedural success, total RF, and procedure time). The secondary endpoint was the recurrence of typical AFL during follow-up. **Results**: In total, 38 patients undergoing temperature-controlled ablation were enrolled and compared to 283 patients undergoing power-controlled ablation. A bidirectional CTI block was achieved in 100% in the temperature-controlled group and 97.5% in the power-controlled group (*p* = 0.7). The total RF time (median: 192 sec (IQR 138–311) vs. 643 sec (IQR 386–1079), *p* < 0.001) and total procedure time (median: 45 min (IQR 34–57) vs. 52 min (IQR 39–70), *p* = 0.01) were shorter with temperature-controlled ablation. At the one-year follow-up, there was no difference in the recurrence of typical AFL between groups. **Conclusions**: Utilization of temperature-controlled ablation for typical AFL increased procedural efficiency with shorter RF and procedure times compared to power-controlled ablation. The recurrence rate of typical AFL after one year was low and did not differ amongst groups.

## 1. Introduction

Typical atrial flutter (AFL) is a common cause of supraventricular tachycardia [1]. Studies estimate the incidence of typical AFL to be about 88/100,000 person-years [2]. The suggested risk factors for typical AFL include male sex, advanced age, heart failure, and chronic obstructive pulmonary disease [2]. Typical AFL is a macro re-entrant circuit around the annulus of the tricuspid valve [3]. Therefore, radiofrequency (RF) ablation of the cavotricuspid isthmus (CTI) and interruption of this circuit is a well-established treatment option for typical AFL [4]. This has led to a steadily increasing number of CTI ablations in the last years. Currently, AFL is the second most common indication for ablation in Switzerland after atrial fibrillation ablation [5]. Due to this trend, new technologies for optimizing the efficiency and efficacy of CTI ablation are being developed.

Open-irrigated power-controlled RF ablation has been established as the standard for most catheter ablations, including CTI ablation. The advantages are less char formation and a deeper lesion size compared to non-irrigated ablation; however, the assessment of tissue temperature is disabled since sensors are located proximal to the irrigation ports [6]. More recently, a novel temperature-controlled RF ablation system using a diamond-tip catheter has been introduced for clinical use [7]. The 4.1 mm tip of this catheter is able to measure surface temperature directly, allowing for temperature-guided ablation instead of power-controlled ablation [7]. Clinical trials to date with this ablation system suggest that the imminent ablation success of this novel catheter is comparable to other commonly used catheters, although RF and procedure durations tend to be shorter [8].

The aim of this study was to compare the procedural efficacy (defined as total RF time, total procedure time, and procedural success), as well as the long-term outcome (recurrence of CTI-dependent AFL during 12 months of follow-up), between the novel temperature-controlled diamond-tip catheter system and an established power-controlled gold-tip catheter system.

## 2. Materials and Methods

### 2.1. Study Group

Data from patients undergoing CTI ablation for typical AFL at the Department of Cardiology, University Hospital Bern, were collected in the prospective (BEAT Flutter) registry, a registry assessing the incidence of AF and AFL after CTI ablation. This database collected procedural data as well as data from recommended follow-up visits after CTI ablation. The inclusion and exclusion criteria of this registry have been published before [9] and are shown in Supplementary Table S1. Data collection and analysis were performed in compliance with study protocols and approved by the local ethics committee.

For the current analysis, consecutive patients undergoing CTI ablation using the novel temperature-controlled diamond-tip catheter (DiamondTemp, Medtronic, Minneapolis, Minnesota) were enrolled. They were compared to a control group of patients who had undergone CTI ablation using a standard power-controlled gold-tip catheter (AlCath Flutter Flux, Biotronik, Berlin, Germany). Patients who had undergone CTI ablation with other ablation catheters and patients with concomitant left atrial ablation procedures such as pulmonary vein isolation were excluded from the analysis.

### 2.2. Baseline Evaluation

At the enrolment visit, trained study personnel obtained baseline patient characteristics, including demographic data, medical history, and typical cardiovascular risk factors. Current symptoms were assessed using the NYHA score [10] for dyspnea and the EHRA score [4] for symptoms of arrhythmia. To assess the risk of stroke and bleeding under oral anticoagulants, the CHA2DS2-VASc and HAS-BLED scores [4] were calculated. If available, the left ventricular ejection fraction (LVEF), LA diameter, and LA volume index were registered. Echocardiography was not routinely performed before ablation, only when otherwise indicated.

### 2.3. Ablation Procedure

A diagnostic catheter (Bard dynamic XT, Boston Scientific, Marlborough, MA, USA) was placed in the coronary sinus. To interrupt the re-entrant circuit of typical AFL, a linear ablation from the cavotricuspid annulus towards the opening of the inferior vena cava was performed. The catheters used for the intervention were either a 4.1 mm temperature-controlled diamond-tip catheter (DiamondTemp, Medtronic) at a temperature setting of 50–60 degrees Celsius or a 3.5 mm power-controlled gold-tip catheter (AlCath Flutter Flux, Biotronik) at a power setting of 30–40 Watts. Both catheters had open irrigation. The procedures were guided by fluoroscopy only. No 3D mapping was used. The procedural endpoint was the documentation of a bidirectional block across the CTI ablation line using differential pacing without evidence of recovery during a 15 min waiting period. The duration of intervention, RF application, and fluoroscopy exposure were collected. The safety endpoint was a composite of pericardial tamponade, an atrioventricular block, and groin complications requiring intervention.

### 2.4. Follow-Up

Patients were recommended to present to their outpatient cardiologist for follow-up after 3, 6, and 12 months to undergo 7-day electrocardiography (ECG) monitoring. The recurrence of typical atrial flutter was considered clinically significant if the duration exceeded 30 s, as recommended by guidelines [1,4]. In case of recurrent symptomatic tachycardia outside of these visits, patients were instructed to obtain 12-lead ECG documentation of the event. Recurrence of typical AFL during follow-up was confirmed by a 7-day ECG, 12-lead ECG, and/or intracardiac ECG during an electrophysiological study. No blanking period was used in this analysis.

### 2.5. Statistical Analysis

Numerical and ordinal variables are shown as medians (interquartile range). Statistical analysis of these variables was performed using the Wilcoxon–Mann–Whitney test. Categorical variables are shown as counts (percentage) and were analyzed using the chi-squared test. Kaplan–Meier curves were constructed to visualize the rate of AFL recurrence in the study groups. These were compared using the log-rank test. A two-sided *p*-value of <0.05 was considered statistically significant. All statistical analysis was performed using R version 4.2.2 [11].

## 3. Results

### 3.1. Patient and Procedural Characteristics

**Figure 1:**
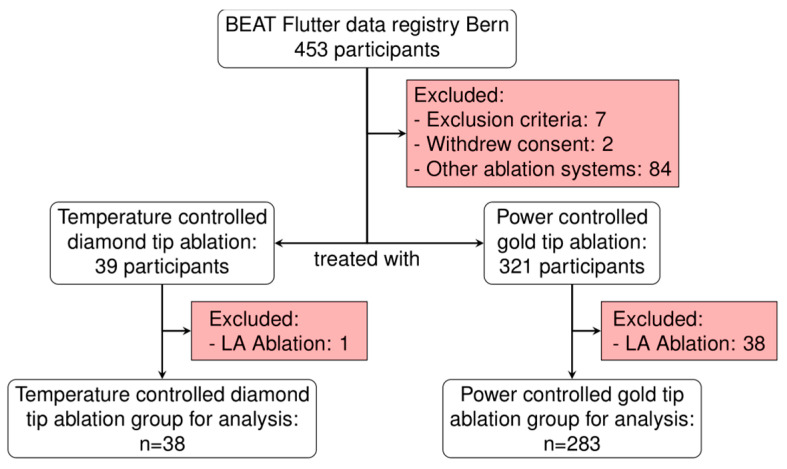
Flowchart showing the patient selection process.

Between 2018 and 2023, 453 patients underwent their first CTI ablation. After the exclusion of 132 patients, 321 remained available for analysis (Figure 1). Of those, 38 patients underwent ablation using the temperature-controlled diamond-tip ablation system and 283 patients underwent ablation with the power-controlled gold-tip ablation system. The baseline characteristics of the patients are shown in Table 1 and showed a balanced distribution between the two groups.

**Figure 2:**
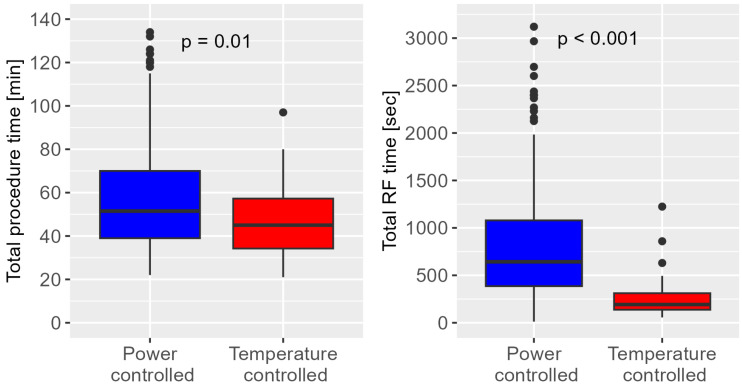
Box plot comparing the procedure time and total radiofrequency (RF) application time between groups.

Procedural data are presented in Table 2. A bidirectional block was achieved in 100% of the temperature-controlled group and in 97.5% of the power-controlled group (*p* = 0.7). The procedure time (45 min (IQR 34–57) vs. 52 min (IQR 39–70); *p* = 0.01) and RF time (192 s (IQR 138–311) vs. 643 s (IQR 386–1079); *p* < 0.001) were significantly shorter using the novel temperature-controlled ablation system (see Figure 2). Regarding fluoroscopy, the radiation dosage was significantly lower in the temperature-controlled group (213 µGy/m^2^ (IQR 98–427) vs. 445 µGy/m^2^ (IQR 193–1162), *p* < 0.001) while fluoroscopy duration was not different between groups. Regarding the primary safety endpoint, no procedural complications occurred in the temperature-controlled ablation group. In the power-controlled group, three events occurred (aneurysma spurium at the puncture site in two patients and a higher-degree atrioventricular block in one patient).

### 3.2. Recurrence of Atrial Flutter

**Figure 3:**
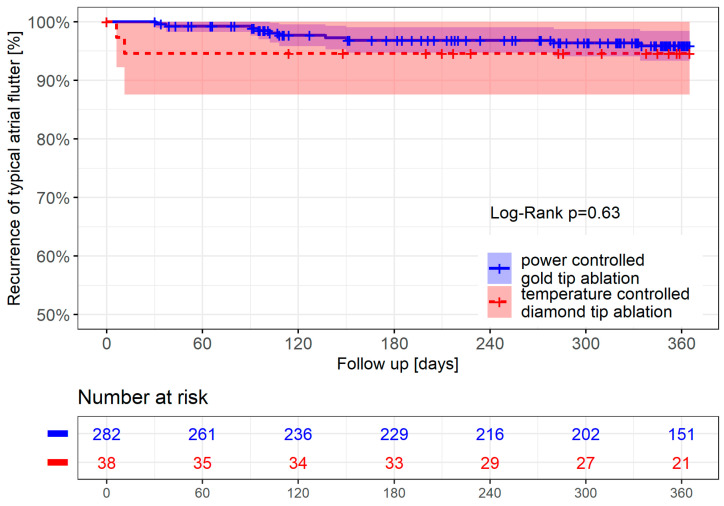
Kaplan–Meier curve showing the survival-free time from recurrence of typical AFL in the temperature-controlled diamond-tip catheter group (red) and the power-controlled gold-tip catheter group (blue).

In the time-to-event analysis, recurrence of typical AFL up to 12 months had occurred in two patients (5.4%) in the temperature-controlled ablation group and ten patients (4.2%) in the power-controlled ablation group (*p* = 0.63 for comparison, Figure 3).

The recurrence of typical AFL in the power-controlled group could be observed for the entire follow-up duration, with a clustering in the first 120 days. All recurrences in the temperature-controlled group occurred within the first 30 days after intervention. Of the 12 patients with a recurrence of typical AFL, re-ablation was performed in eight patients (one in the temperature-controlled group and seven in the power-controlled group). A recovery of the CTI block was documented for all cases.

## 4. Discussion

This study aimed to further investigate and compare a novel temperature-controlled diamond-tip ablation system to an established power-controlled gold-tip ablation system. Our key findings are as follows: (1) Acute procedural success, defined as a bidirectional CTI block, was achieved in nearly all patients in both groups. (2) The temperature-controlled ablation system showed a significantly shorter procedure duration, a lower total RF time, and a lower radiation dosage when compared to a conventional power-controlled system. (3) The recurrence rates of typical AFL after 1 year were low and comparable among the ablation systems.

Regarding intervention times, our results are in line with previous research investigating the novel ablation system for CTI ablation or pulmonary vein isolation (PVI) [12,13,14]. The main hypothesis explaining the shorter procedure duration and RF ablation time is the utilization of high-power short-duration ablation. This method leads to a steeper temperature gradient in deeper tissues and is useful for creating shallow lesions in areas with thin myocardium, such as the atria [15]. Ex vivo studies analyzing lesion creation using the temperature-controlled system showed clinically sufficient lesion depth using single RF applications of about 15 s [7,16]. Therefore, the total RF time and energy are lower when comparing the temperature-controlled system to the established catheters, which also translates into a shorter total procedure time. Another postulated hypothesis for the shorter procedure time is the tip’s ability to record high-resolution electrograms, allowing for more accurate monitoring of lesion generation and gap identification [8,13].

The sole discrepancy when comparing the results of previous studies [8,12,13,14] is in regard to fluoroscopy duration. Some studies were able to show a decrease in fluoroscopy duration and radiation dosage when using the novel ablation system [8], while others were not able to show a difference [14]. Our results were only able to show a reduction in the total radiation dosage and not fluoroscopy duration. Due to the small sample size of the temperature-controlled group, it is prone to different factors influencing fluoroscopy duration, incidentally raising it. Different settings used for fluoroscopy, such as frame rate, field of view, and other slight variations between operators, could explain these findings. Another factor influencing fluoroscopy during ablation is the higher body mass index (BMI) of the temperature-controlled group, although this was not statistically significant. It has been shown that BMI can influence fluoroscopy duration, as well as the radiation dosage of ablation procedures [17]. Further research with a larger study population is required to more accurately assess fluoroscopy use when comparing temperature-controlled systems to power-controlled systems.

Acute CTI ablation success is measured by showing a bidirectional block of the CTI ablation line [18]. Using the novel ablation system, all interventions ended with evidence of a bidirectional block. In comparison, 7/283 (2.5%) patients in the control group did not meet this criterion after ablation. Reasons for not achieving bidirectional block were not examined. These results are in line with the study by Ramak et al. [8], who reported achieving a bidirectional block in all 15 of their patients treated with the temperature-controlled ablation system.

Regarding the early recurrence of typical AFL after CTI ablation, no blanking period is used in most cases. Due to the strict dependency of typical AFL upon the CTI, only reconnection or insufficient ablation can cause recurrence. A main risk factor of typical AFL recurrence is ablation duration, which most likely corresponds to difficulty in achieving a CTI block [19]. This is in contrast to AF ablation and recurrence thereof. The ablation lesions, local tissue damage, and surrounding inflammation can cause early episodes of AF after ablation, which subside after healing and scar formation, explaining the need for a blanking period to assess AF recurrence after ablation [20].

To the best of our knowledge, this is the first study comparing the recurrence rate of typical AFL 1 year after ablation of the CTI between the novel temperature-controlled ablation system and an established power-controlled ablation system. Current guidelines suggest a recurrence rate of typical AFL after CTI ablation of <10% [1,21]. Both systems achieved comparable long-term ablation success, with recurrence rates lower than those suggested by guidelines. Due to the limited number of ablations performed with the novel system in our center, the temperature-controlled group is underpowered to confirm or exclude marginal differences in the recurrence rate compared to the power-controlled group.

When examining the use of a novel catheter, some limitations of our database arise. The first is the small sample size of the intervention group. This analysis only included our first experience with using the novel catheter, which explains the sample size. This limits the significance of our statistical findings until further research using larger samples is available. During the collection of these intervention cases, a much larger number of control cases were included in the database. We decided to compare our small intervention group to this larger control group in order to compare the novel catheter to our daily practice. Matching was not performed due to the two groups not being statistically different in the baseline analysis. Second, the parameters collected during the intervention are not as detailed as in other trials. For example, the waiting time until the measurement of the bidirectional block was not investigated. Third, in the 1-year follow-up period, patients and their outpatient physicians received the recommendation to monitor for the recurrence of arrhythmia with Holter ECGs after 3, 6, and 12 months, as well as in the case of symptomatic arrhythmias. Due to the observational nature of this study, some of those ECGs were missing. Given that typical AFL is mostly a persistent and not a paroxysmal arrhythmia, we do not believe that the follow-up strategy resulted in the significant under-reporting of AFL recurrences. Fourth, the distinction of typical vs. atypical flutter on a Holter ECG, as well as on a 12-lead ECG, can be challenging. While a 12-lead ECG can be highly suggestive of typical AFL and reconnection of the CTI, this cannot be confirmed unless an electrophysiological study is performed [22]. Accordingly, recurrent AFL episodes were classified as a recurrence of typical AFL unless a repeat procedure had shown documentation of a persistent CTI Block (in which case the AFL recurrence was classified as an atypical AFL).

Currently, multiple ablation systems exist on the market for performing high-power short-duration RF ablation. Initial research suggests that in most indications, when compared to standard RF ablation, these systems are not inferior in terms of clinical outcomes and periprocedural complications, yet they generate lesions faster and shorten procedure duration [23,24,25]. In recent years, different catheter systems have been developed, using different approaches to control and titrate the RF output (power-controlled versus temperature-controlled) and using different materials with different conductive properties for the catheter tips (gold vs. diamond). Accordingly, there are now several combinations and options to choose from in the electrophysiology lab. Further research directly comparing these methods is needed to define the future standard for radiofrequency ablation.

## 5. Conclusions

The use of a temperature-controlled diamond-tip catheter system for ablation of typical AFL resulted in increased procedural efficiency with shorter RF and procedure times compared to power-controlled ablation with a gold-tip catheter. Outcomes after 1 year were not different between both groups, with low rates of atrial flutter recurrences.

## Figures and Tables

**Table 1:** Baseline characteristics and echocardiography data at inclusion. Numerical and ordinal variables are shown as the median (interquartile range). Categorical variables are shown as counts (percentage). *p*-values were obtained using the Wilcoxon–Mann–Whitney test for numerical and ordinal variables and the chi-squared test for categorical variables. Abbreviations: BMI = body mass index; CHA2DS2-VASc Score = Congestive heart failure, Hypertension, Age, Diabetes, Stroke, Vascular disease, Age, Sex category score; HAS-BLED Score = Hypertension, Abnormal liver/renal function, Stroke history, Bleeding predisposition, Labile INR, Elderly, Drug/alcohol usage score; EHRA Score = European heart rhythm association score; NYHA Score = New York heart association score; LVEF = left ventricular ejection fraction; LV = left ventricle; LA = left atrium.

	Temperature-Controlled Diamond-Tip Catheter (n = 38)	Power-Controlled Gold-Tip Catheter (n = 283)	*p*-Value
Age, years	68.5 (64–76)	71 (63–76)	0.56
BMI, kg/m^2^	26.4 (23–30)	27.4 (24–31)	0.33
Sex, female	5 (13.2)	59 (20.8)	0.37
Coronary artery disease, n	11 (28.9)	71 (25.4)	0.68
Tachycardiomyopathy, n	5 (13.2)	20 (7.2)	0.33
Prior heart failure, n	10 (26.3)	65 (23.0)	0.80
Prior atrial fibrillation, n	17 (44.7)	85 (30.4)	0.11
Hypertension, n	20 (52.6)	177 (62.5)	0.32
Diabetes, n	8 (21.1)	62 (21.9)	1.00
Scores			
CHA2DS2-VASc Score	3 (1.25–4)	3 (1.5–4)	0.94
HAS-BLED Score	2 (1–3)	2 (1–3)	0.21
EHRA score	2 (2–2)	2 (2–3)	0.15
NYHA score	1 (1–2)	2 (1–2)	0.26
Echocardiography Data			
LVEF, %	60 (45–65)	55 (45–60)	0.11
LA diameter, mm	43.5 (42–51)	43 (40–48)	0.31
LA volume index, mL/m^2^	40 (35–48)	41 (34–50)	0.87
Treatment			
Antiarrhythmic drug, n	7 (18.4)	51 (18.0)	1.00
Beta-blockers, n	25 (65.8)	210 (74.2)	0.37
Oral anticoagulants, n	36 (94.7)	248 (87.6)	0.31
Oral antiplatelet therapy, n	3 (7.9)	36 (12.7)	0.55

**Table 2:** Procedural data for CTI ablations. Numerical variables are shown as medians (interquartile range). Categorical variables are shown as counts (percentage). *p*-values were obtained using the Wilcoxon–Mann–Whitney test for ordinal variables and the chi-squared test for categorical variables. Abbreviations: CTI = cavotricuspid isthmus; RF = radiofrequency.

	Temperature-Controlled Diamond-Tip Catheter(n = 38)	Temperature-Controlled Diamond-Tip Catheter (n = 38)	*p*-Value
Procedure duration, min	45 (34–57)	52 (39–70)	0.01
RF time for CTI ablation, s	192 (138–311)	643 (386–1079)	<0.001
Radiation dose, µGy/m^2^	213 (98–427)	445 (193–1162)	<0.001
Fluoroscopy time, min	13.3 (7.1–20.9)	11.4 (7.5–17.4)	0.83
Confirmed bidirectional block, n	38 (100)	276 (97.5)	0.70

## Data Availability

The data presented in this study are available on request from the corresponding author. (please specify the reason for restriction, e.g., the data are not publicly available due to privacy or ethical restrictions.)

## Supplementary Materials

The following supporting information can be downloaded at: https://www.mdpi.com/article/10.3390/jcm14030701/s1, Table S1: Inclusion and exclusion criteria of the BEAT Flutter data registry.

## References

[1] Brugada J., Katritsis D.G., Arbelo E., Arribas F., Bax J.J., Blomström-Lundqvist C., Calkins H., Corrado D., Deftereos S.G., Diller G.-P. (2019). 2019 ESC Guidelines for the Management of Patients with Supraventricular Tachycardia. The Task Force for the Management of Patients with Supraventricular Tachycardia of the European Society of Cardiology (ESC): Developed in Collaboration with the Association for European Paediatric and Congenital Cardiology (AEPC). Eur. Heart J..

[2] Granada J., Uribe W., Chyou P.-H., Maassen K., Vierkant R., Smith P.N., Hayes J., Eaker E., Vidaillet H. (2000). Incidence and Predictors of Atrial Flutter in the General Population. J. Am. Coll. Cardiol..

[3] Waldo A.L., Feld G.K. (2008). Inter-Relationships of Atrial Fibrillation and Atrial Flutter: Mechanisms and Clinical Implications. J. Am. Coll. Cardiol..

[4] Hindricks G., Potpara T., Dagres N., Arbelo E., Bax J.J., Blomström-Lundqvist C., Boriani G., Castella M., Dan G.-A., Dilaveris P.E. (2020). 2020 ESC Guidelines for the Diagnosis and Management of Atrial Fibrillation Developed in Collaboration with the European Association for Cardio-Thoracic Surgery (EACTS): The Task Force for the Diagnosis and Management of Atrial Fibrillation of the European Society of Cardiology (ESC) Developed with the Special Contribution of the European Heart Rhythm Association (EHRA) of the ESC. Eur. Heart J..

[5] Molitor N., Yalcinkaya E., Auricchio A., Burri H., Delacretaz E., Kühne M., Menafoglio A., Reek S., Reichlin T., Herrera-Siklody C. (2021). Swiss National Registry on Catheter Ablation Procedures: Changing Trends over the Last 20 Years. J. Clin. Med..

[6] Waldo A.L., Wilber D.J., Marchlinski F.E., Stevenson W.G., Aker B., Boo L.M., Jackman W.M. (2012). Safety of the Open-Irrigated Ablation Catheter for Radiofrequency Ablation: Safety Analysis from Six Clinical Studies. Pacing Clin. Electrophysiol..

[7] Verma A., Schmidt M.M., Lalonde J.-P., Ramirez D.A., Getman M.K. (2021). Assessing the Relationship of Applied Force and Ablation Duration on Lesion Size Using a Diamond Tip Catheter Ablation System. Circ. Arrhythm. Electrophysiol..

[8] Ramak R., Lipartiti F., Mojica J., Monaco C., Bisignani A., Eltsov I., Sorgente A., Capulzini L., Paparella G., Deruyter B. (2022). Comparison between the Novel Diamond Temp and the Classical 8-Mm Tip Ablation Catheters in the Setting of Typical Atrial Flutter. J. Interv. Card. Electrophysiol..

[9] Krisai P., Roten L., Zeljkovic I., Pavlovic N., Ammann P., Reichlin T., Auf der Maur E., Streicher O., Knecht S., Kühne M. (2021). Prospective Evaluation of a Standardized Screening for Atrial Fibrillation after Ablation of Cavotricuspid Isthmus Dependent Atrial Flutter. J. Clin. Med..

[10] McDonagh T.A., Metra M., Adamo M., Gardner R.S., Baumbach A., Böhm M., Burri H., Butler J., Čelutkienė J., Chioncel O. (2021). 2021 ESC Guidelines for the Diagnosis and Treatment of Acute and Chronic Heart Failure: Developed by the Task Force for the Diagnosis and Treatment of Acute and Chronic Heart Failure of the European Society of Cardiology (ESC) With the Special Contribution of the Heart Failure Association (HFA) of the ESC. Eur. Heart J..

[11] R Core Team (2022). R: A Language and Environment for Statistical Computing.

[12] Dittrich S., Braun M., Bergau L., Sohns C., Sultan A., Lüker J., Wörmann J., Scheurlen C., Schipper J.-H., van den Bruck J.-H. (2023). Early Real-World Experience Using Temperature-Guided Diamond Tip Facilitated High-Power Ablation for Catheter Ablation of Atrial Fibrillation. J. Interv. Card. Electrophysiol..

[13] Iwasawa J., Koruth J.S., Petru J., Dujka L., Kralovec S., Mzourkova K., Dukkipati S.R., Neuzil P., Reddy V.Y. (2017). Temperature-Controlled Radiofrequency Ablation for Pulmonary Vein Isolation in Patients With Atrial Fibrillation. J. Am. Coll. Cardiol..

[14] Kautzner J., Albenque J.-P., Natale A., Maddox W., Cuoco F., Neuzil P., Poty H., Getman M.K., Liu S., Starek Z. (2021). A Novel Temperature-Controlled Radiofrequency Catheter Ablation System Used to Treat Patients With Paroxysmal Atrial Fibrillation. JACC Clin. Electrophysiol..

[15] Calkins H., Hindricks G., Cappato R., Kim Y.-H., Saad E.B., Aguinaga L., Akar J.G., Badhwar V., Brugada J., Camm J. (2017). 2017 HRS/EHRA/ECAS/APHRS/SOLAECE Expert Consensus Statement on Catheter and Surgical Ablation of Atrial Fibrillation. Heart Rhythm.

[16] Sasaki W., Matsumoto K., Higuchi S., Mori H., Fukaya H., Kawano D., Tanaka N., Narita M., Tsutsui K., Ikeda Y. (2023). Detailed Analysis of the Lesion Formation Using a Diamond Tip Catheter in an Ex Vivo Experimental Model. J. Cardiol..

[17] Schenker N., Von Blumenthal F., Hakmi S., Lemes C., Mathew S., Rottner L., Wohlmuth P., Reißmann B., Rillig A., Metzner A. (2022). Impact of Obesity on Acute Complications of Catheter Ablation for Cardiac Arrhythmia. J. Cardiovasc. Electrophysiol..

[18] Schwartzman D., Callans D.J., Gottlieb C.D., Dillon S.M., Movsowitz C., Marchlinski F.E. (1996). Conduction Block in the Inferior Vena Caval-Tricuspid Valve Isthmus: Association with Outcome of Radiofrequency Ablation of Type I Atrial Flutter. J. Am. Coll. Cardiol..

[19] Calkins H., Canby R., Weiss R., Taylor G., Wells P., Chinitz L., Milstein S., Compton S., Oleson K., Sherfesee L. (2004). Results of Catheter Ablation of Typical Atrial Flutter. Am. J. Cardiol..

[20] Tzeis S., Gerstenfeld E.P., Kalman J., Saad E.B., Sepehri Shamloo A., Andrade J.G., Barbhaiya C.R., Baykaner T., Boveda S., Calkins H. (2024). 2024 European Heart Rhythm Association/Heart Rhythm Society/Asia Pacific Heart Rhythm Society/Latin American Heart Rhythm Society Expert Consensus Statement on Catheter and Surgical Ablation of Atrial Fibrillation. Europace.

[21] Schmieder S., Ndrepepa G., Dong J., Zrenner B., Schreieck J., Schneider M.A.E., Karch M.R., Schmitt C. (2003). Acute and Long-Term Results of Radiofrequency Ablation of Common Atrial Flutter and the Influence of the Right Atrial Isthmus Ablation on the Occurrence of Atrial Fibrillation. Eur. Heart J..

[22] Frisch D.R., Frankel E., Gill D., Danaf J.A. (2021). Algorithm for Cavo-Tricuspid Isthmus Flutter on Surface ECGs: The ACTIONS Study. Open Heart.

[23] Dello Russo A., D’Angelo L., Compagnucci P., Cipolletta L., Parisi Q., Valeri Y., Campanelli F., Volpato G., Carboni L., Ciliberti G. (2023). High-Power Short-Duration Catheter Ablation of Atrial Fibrillation: Is It Really a New Era? Comparison between New and Old Radiofrequency Contact Force–Sensing Catheters. J. Interv. Card. Electrophysiol..

[24] Dello Russo A., Compagnucci P., Bergonti M., Cipolletta L., Parisi Q., Volpato G., Santarelli G., Colonnelli M., Saenen J., Valeri Y. (2023). Microelectrode Voltage Mapping for Substrate Assessment in Catheter Ablation of Ventricular Tachycardia: A Dual-center Experience. J. Cardiovasc. Electrophysiol..

[25] Compagnucci P., Volpato G., Cipolletta L., Parisi Q., Valeri Y., Campanelli F., D’Angelo L., Ciliberti G., Stronati G., Carboni L. (2024). Posterior wall ablation for persistent atrial fibrillation: Very-high-power short-duration versus standard-power radiofrequency ablation. Heart Rhythm O2.

