# Supplementary material for: Comparison of a Novel Temperature-Controlled Diamond-Tip Catheter and a Power-Controlled Gold-Tip Catheter for the Irrigated Ablation of Cavotricuspid Isthmus-Dependent Atrial Flutter
Source: J Clin Med. 2025 Jan 22;14(3):701. doi: 10.3390/jcm14030701 (PMC11818538; doi:10.3390/jcm14030701)
Supplement: Supplementary file 1 [file jcm-14-00701-s001.zip › jcm-3399940-supplementary.pdf]

Supplemental Table 1: Inclusion and exclusion criteria of the BEAT Flutter data registry	
- CTI ablation<br>- Typical atrial flutter	- Missing informed consent<br>- Atypical atrial flutter (CTI independent)<br>- Prior heart surgery<br>- Congenital heart anomaly
